# Comparison of bovine leukemia virus (BLV) and CMV promoter-driven reporter gene expression in BLV-infected and non-infected cells

**DOI:** 10.1186/1479-0556-2-11

**Published:** 2004-08-24

**Authors:** Jerome S Harms, Kurt A Eakle, Lillian S Kuo, Robert D Bremel, Gary A Splitter

**Affiliations:** 1 Department of Animal Health and Biomedical Sciences, University of Wisconsin-Madison, Madison, WI 53706-1581, USA; 2 GALA Biotech, 8137 Forsythia Street, Middleton, WI 53562, USA; 3 IoGenetics LLC, 3591 Anderson St., Suite 218, Madison, WI 53704, USA

## Abstract

**Background:**

Viral promoters are used in mammalian expression vectors because they generally have strong activity in a wide variety of cells of differing tissues and species.

**Methods:**

The utility of the BLV LTR/promoter (BLVp) for use in mammalian expression vectors was investigated through direct comparison to the CMV promoter (CMVp). Promoter activity was measured using luciferase assays of cell lines from different tissues and species stably transduced with BLVp or CMVp driven luciferase vectors including D17, FLK, BL3.1 and primary bovine B cells. Cells were also modified through the addition of BLV Tax expression vectors and/or BLV infection as well as treatment with trichostatin A (TSA).

**Results:**

Results indicate the BLV promoter, while having low basal activity compared to the CMV promoter, can be induced to high-levels of activity similar to the CMV promoter in all cells tested. Tax or BLV infection specifically enhanced BLVp activity with no effect on CMVp activity. In contrast, the non-specific activator, TSA, enhanced both BLVp and CMVp activity.

**Conclusion:**

Based on these data, we conclude the BLV promoter could be very useful for transgene expression in mammalian expression vectors.

## Background

Viral promoters are commonly used as regulatory elements in gene therapy vectors due to their strong activity in various cell lines in vitro. Probably the most widely used promoter in mammalian expression systems is the human cytomegalovirus immediate-early gene (CMV) promoter. The CMV promoter induces high-level constitutive expression in a variety of mammalian cell lines [1]. In many gene therapy applications, however, an inducible or cell specific promoter would be more appropriate. A regulated transgene expression system in mammalian cells is preferable for effective and safe gene therapy and for the study of gene function in cell biology. The most important features of an inducible promoter would be 1) low basal expression levels; 2) high induced expression; and 3) inducer-specific, modulated expression [2]. Our need for a mammalian expression vector promoter for preventative gene therapy that would be induced by bovine leukemia virus (BLV) infection or BLV Tax protein expression led us to investigate the use of the BLV promoter for gene therapy.

The U3 region of the BLV promoter, located in the 5' long terminal repeat (LTR), contains several important *cis*-acting elements in addition to the CAAT box, TATA box, and transcription start site [3,4]. The major regulatory elements are three copies of an imperfectly conserved 21-bp sequence called the tax responsive element (TxRE). The TxREs are essential for the promoter's responsiveness to the Tax transactivator protein encoded by the 3' end of the proviral genome [5]. These *cis*-elements contain motifs resembling the cyclic AMP-responsive element (CRE) as well as an E box sequence [6]. Tax does not bind directly to the TxRE but interacts with cellular proteins that recognize the CRE including the transcription factors CREB, ATF-1, and ATF-2 [7-9]. The transcription factor AP4 can potentially bind to the E box sequence and is important in Tax activation [10]. There is a glucocorticoid responsive element (GRE) that responds to dexamethasone in the presence of glucocorticoid receptors and Tax [11]. A nuclear factor κB (NFκB) binding site responds to phorbol 12-myristate 13-acetate (PMA) treatment [12]. Finally, there is a Tax transactivator independent site specific for the B cell transcription factors PU.1 and Spi-B [13]. This PU.1/Spi-B binding site may be involved in the B lymphocyte tropism of BLV.

We hypothesized that the BLV promoter could be used in mammalian expression vectors for regulated high-level gene expression. Our approach was to compare reporter gene expression driven by either the BLV promoter or CMV promoter in different cell types with or without BLV infection or Tax induction. Our results demonstrate that the BLV promoter can be induced to express the reporter gene to levels as great as the constitutive CMV promoter.

## Methods

### Cell culture

All cells used in these studies were maintained in RPMI 1640 medium (Invitrogen) supplemented with 10% fetal calf serum (FCS), 4.5% dextrose, 1 mM sodium pyruvate, and antibiotic-antimycotic solution (100 μg/ml penicillin G sodium, 100 μg/ml streptomycin sulfate, 0.25 μg/ml amphotericin B). In addition, the following concentrations of drugs were added for each selective media: Blasticidin-S (Invivogen) 10 μg/ml; G418-sulfate (Alexis Biochemical) 400 μg/ml. The following cell lines were used: D17 [dog osteosarcoma; ATCC CCL-183; [14]], FLK [sheep kidney; BLV expresser; [15]], BL3.1 [bovine B-lymphosarcoma; BLV expresser; ATCC CRL-2306; [16]]. Primary bovine B cells were supplemented with 10 ng/ml each of recombinant human interleukin-4 and interleukin-7 (Peprotech, Inc.), and gamma-irradiated (4,000 R) murine CD40L-expressing L cells (J558L; a gift from Philip Griebel) as described elsewhere [17]. For the TSA experiments, Trichostatin A (Sigma) was supplemented at 500 nM for 48 h. Cells were cultured at 37°C in a 5% CO2 humidified atmosphere. Viable cells were identified by trypan blue dye exclusion, and cell number was counted with a hemacytometer.

Primary bovine B cells were purified as follows. Peripheral blood mononuclear cells (PBMC) were isolated from heparinized cow blood through a ficoll density gradient as previously described [18]. B cells were separated from the PBMCs using the MiniMACS system following the manufacturer's (Miltenyi Biotec) protocol. Briefly, 1 × 10^7 ^cells were stained for 15 min at 6° – 12°C with 10 μg/100 μl total volume anti-IgM (PIG45A; VMRD, Inc.). After washing, 20 μl/100 μl total volume of MACS rat anti-mouse IgG2a+b microbeads were mixed with the cells and incubated for 15 min at 6° – 12°C. Cells were thoroughly washed, and magnetically separated. These IgM+ cells were considered primary B cells. Microfluorimetry using anti-IgM (PIG45A; VMRD, Inc.) indicated 90% purity. Stably transduced cell lines were generated after one week in selective media. Primary B cells were analyzed after one week in selective media since they began to die out after two weeks in culture.

### Vector construction

The plasmid pBLV913 (a gift from David Derse), coding for an infectious molecular clone of BLV [5] was used as the source for the BLV promoter and BLV Tax sequences. Briefly, the BLV promoter from the U3 region of 3' LTR of BLV was isolated from plasmid pBLV913 (Derse) as a 345 bp fragment (GenBank LOCUS BLVCG, ACCESSION K02120 bp 8096 – 8440) and cloned in place of the CMV promoter fragment into pLNCX (Clontech; Genebank LOCUS SYNMMLPLN3 ACCESSION M28247 – CMV promoter removed as *Bam *HI-*Hind *III fragment) to create the vector pLNBlv. The pLNBlv and pLNCX retrovector plasmids were modified to place the Gateway Rfa cassette (1.7 kb; Invitrogen) downstream of the internal promoters (BLV or CMV) in order to simplify further cloning, to create retrovector plasmids pLNBlv-G or pLNC-G. For enhanced protein expression, the WPRE element (from plasmid BluescriptII SK+ WPRE-B11 (a gift from Tom Hope–the same as bp 2717–3309 of Genbank Locus OHVHEPBA ACCESSION J04514) was cloned downstream of the Gateway Rfa cassette with standard cloning methods to create vectors pLNC-GW and pLNBlv-GW. The source for firefly luciferase encoding sequence was pGEM-luc (Promega). The luciferase coding sequence was subcloned into pENTR1A (Invitrogen) to engineer the Gateway entry vector pENTR1A/luc. The Luciferase gene was recombined into pLNC-GW or pLNBlv-GW using LR Clonase (Invitrogen) per manufacturer's instructions. The promoter-less luciferase expression control vector pLN[]W/luc was engineered by removing the BLV promoter (*Bam *HI digest) from pLNBlv/luc. BLV Tax (Genbank Locus AAF97920) was isolated by reverse transcription PCR from FLK cells and subcloned into pENTR1A (Invitrogen) to engineer the Gateway entry vector pENTR1A/Tax. The Tax gene was recombined into pLBC-GW where the neomycin resistance gene of pLNC-GW was replaced with the blasticidin resistance gene.

Throughout these studies we assayed expression vectors with and without the WPRE. WPRE enhanced transgene expression in all cell lines used, and in a promoter-independent fashion (about 2-fold greater for BLVp and CMVp in D17 cells). Subsequently, all data shown in this report are only with vectors containing WPRE.

### Cell transfection and transduction

Retrovirus-mediated gene transfer was accomplished using the BD Retro-X System (BD Biosciences Clontech) following the manufacturer's suggested protocol. Briefly, 100 mm × 20 mm tissue culture dishes (Falcon) were seeded with the packaging cell line GP2-293 at 70–90% confluency. Each dish of GP2-293 cells was co-transfected with 5 μg each of retroviral vector and the envelope glycoprotein expression vector pVSV-G using 15 μl/transfection of Lipofectamine 2000 (Invitrogen) cationic lipid reagent for 3 h in a total volume of 5 ml medium/dish. Subsequently, transfection medium was replaced with 10 ml growth medium, and the cells were incubated for 72 h. Retrovirus-containing supernatant was then harvested and passed through a 0.45 μm cellulose acetate filter, then concentrated by ultracentrifugation at 50,000 × g for 30 min at 4°C. Supernatant was carefully poured off and virus was resuspended in the residue (~200 μl) and frozen (-70°C) for future use. Cells for transduction were plated on 6-well tissue culture plates (Falcon) at 50% confluency. Concentrated retrovirus (titer unknown) along with polybrene (8 μg/ml) were added to one ml/well cells (in a 6-well plate) and incubated overnight. Transduction medium was replaced with fresh growth medium, and the following day cells were split into appropriate selective medium. BLV was harvested from supernatant of FLK cells, concentrated, and used to transduce cells in a similar fashion.

### Luciferase assay

Luciferase assays (Promega) were performed using a single-tube luminometer (Pharmingen) to measure relative light units (RLU) on a linear scale. Cells to be assayed were counted using a hemacytometer, and 1 × 10^6 ^cells were aliquoted to 1.5 ml microcentrifuge tubes. Then, cells were pelleted at 300 × g for 10 min, washed once with PBS, and lysed with 200 μl reporter lysis buffer (Promega). Lysate was stored at -20°C until assayed. Lysate was thawed and pelleted (300 × g for 10 min), and luciferase was measured with the luminometer using 10 μl lysate/50 μl reagent for 10 s. Linear range was under 1 × 10^7 ^RLU.

### Statistical analysis

Student's t-test was performed for statistical evaluation of the results. Results are expressed as the arithmetic mean with the variance of the mean (mean ± SE).

## Results

### The BLV promoter was engineered to drive reporter genes

**Figure 1:**
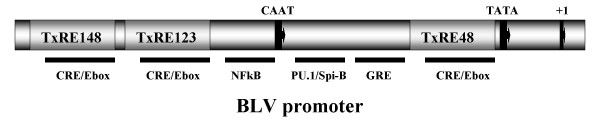
Schematic representation of BLV promoter used in comparison studies. The BLV promoter (BLVp) consisting of the U3 region of the 5'LTR of BLV includes the basic elements of transcription start site (+1), CAAT (nt -97/-92) and TATA (nt -43/-37) boxes as shown. Unique to the BLVp are the three imperfectly conserved 21 bp sequences known as the Tax Responsive Elements (TxRE). The numbers following the TxRE designation represent its position relative to the transcription start site. Each TxRE contains a consensus E box-binding motif overlapping an imperfect cyclic AMP responsive element motif (CRE/Ebox). Additionally, the BLVp contains a glucocorticoid responsive element (GRE), Nuclear Factor Kappa Binding motif (NFkB), and B cell specific PU.1 or Spi-B transactivator binding motif (PU.1/Spi-B). The transcription elements are not drawn to scale.

Our studies utilized a commercially available retroviral system with its standard CMV promoter (CMVp) or replaced with the BLV promoter (BLVp). Figure 1 shows a schematic of the BLV promoter used in these studies with its unique regulatory elements. The luciferase reporter gene was used to compare promoter expression strength within different cell lines and treatments. The Woodchuck Hepatitis Virus posttranscriptional regulatory element (WPRE) was also incorporated to enhance transgene expression within these retroviral vectors [19,20]. WPRE has been reported to significantly stimulate expression of transgenes in a promoter-independent fashion [19]. Retroviral vectors were used because of the ease of stable cell line establishment, and because of their prominent use in transgenics and gene therapy. The commercially available retroviral vector used in these studies contained the CMV IE promoter for transgene expression. We modified this retrovector for comparison studies replacing the CMV IE promoter with the BLV promoter (see methods). Cells of several different tissues and species were used in our studies.

### The BLV promoter can be as strong as the CMV promoter depending on the host cell

**Figure 2:**
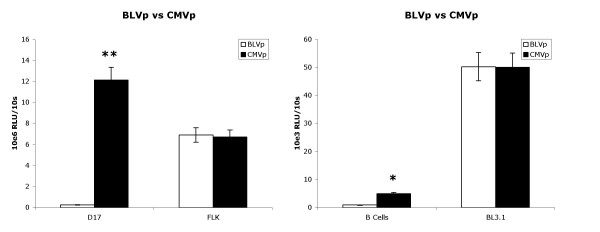
BLVp and CMVp activity comparison in D17, FLK, primary cow B cells, and BL3.1. Relative light units (RLU) of luciferase activity driven by either the BLV promoter (BLVp) or CMV promoter (CMVp) of 1 × 10^6 ^stably transduced cells was measured during a 10 s period. Bars represent the arithmetic mean and variance of 10 experiments. *P < 0.05; **P < 0.001 determined by t-test.

In contrast to the constitutive expression of the CMV promoter, the BLV promoter has *cis *elements that are dependent on BLV Tax for transgene expression [5,21,22]. We hypothesized therefore that in a cell line such as D17, the BLV promoter would have little or no activity compared to the CMV promoter. Conversely, in a cell line expressing the BLV Tax transactivator such as the BLV-producing FLK cell line, the BLV promoter would have similar activity compared to the CMV promoter. We tested this assumption with luciferase as the transgene and found indeed, BLV promoter activity was about 50-fold less than CMV promoter activity in D17 cells but was about equal in FLK cells (Fig. 2). As shown in Figure 1, the BLVp also has a *cis *element that is B cell specific (PU.1/Spi-B). We therefore compared the strengths of BLV and CMV promoters in primary B cells and a BLV infected B cell line hypothesizing that BLVp expression would be comparable to CMVp activity. BLVp activity was still less than CMVp activity in primary B cells but by only about a 5-fold difference (Fig. 2). In the BLV infected BL3.1 cell line, BLVp activity was approximately equal to CMVp activity, analogous to results using the BLV infected FLK cell line. Thus, the BLV promoter can be as strong as the CMV promoter within a cell line under specific conditions e.g. BLV infection/Tax expression.

### BLV infection enhances BLV promoter expression but has no effect on the CMV promoter

**Figure 3:**
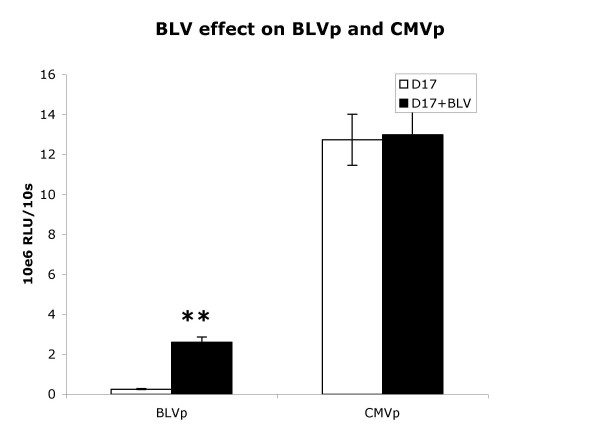
BLV infection enhances BLVp activity but has no effect on CMVp activity. D17 cells or D17 cells infected with and productively expressing BLV (D17+BLV) were transduced with luciferase expression vectors. Relative light units (RLU) of luciferase activity driven by either the BLV promoter (BLVp) or CMV promoter (CMVp) of 1 × 10^6 ^stably trasduced cells was measured during a 10 s period. Bars represent the arithmetic mean and variance of 10 experiments. **P < 0.001 determined by t-test.

Since BLV promoter activity was greater than CMV promoter activity in the BLV infected FLK cell line but minimal compared to CMV promoter activity in the non-BLV infected D17 cell line, we set out to determine whether BLV infection of D17 cells would enhance BLVp and/or suppress CMVp expression. The dog derived D17 cell line can be infected with BLV albeit not very efficiently [14]. D17 cells were infected with concentrated BLV from FLK cells, then clonally selected for BLV expression using *pol *RT-PCR and BLV reverse transcriptase assay of the supernatant (data not shown). Luciferase assays demonstrated that BLV promoter activity in infected D17 cells was about 10-fold greater than BLV promoter activity in non-infected D17 cells (Fig. 3). CMV promoter activity remained unchanged.

### BLV Tax enhances BLV promoter expression but has no effect on the CMV promoter

**Figure 4:**
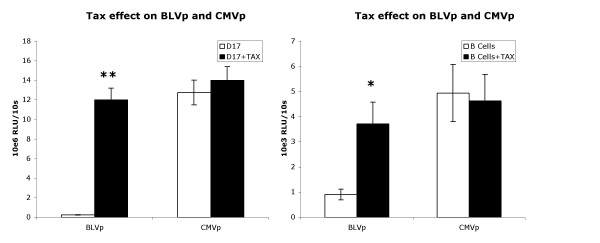
BLV Tax expression significantly enhances BLVp activity but has no effect on CMVp activity. D17 cells and primary bovine B cells (D17; B cells), or D17 cells and primary bovine B cells stably transduced with a BLV Tax expression vector (D17+TAX; B cells+TAX), were assayed. Relative light units (RLU) of luciferase activity driven by either the BLV promoter (BLVp) or CMV promoter (CMVp) of 1 × 10^6 ^stably transduced cells were measured during a 10 s period. Bars represent the arithmetic mean and variance of 10 experiments. **P < 0.001 determined by t-test.

**Figure 5:**
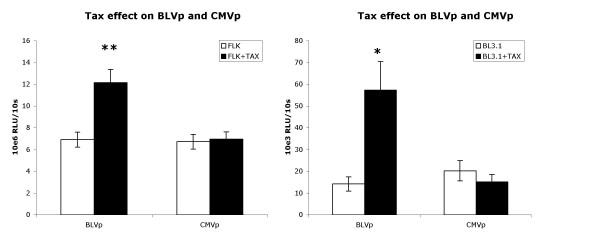
Tax trans-gene expression significantly enhances BLVp activity in cells producing high levels of BLV. FLK and BL3.1 cells, or FLK and BL3.1 cells stably transduced with a BLV Tax expression vector (FLK+TAX; BL3.1+TAX) were assayed. Relative light units (RLU) of luciferase activity driven by either the BLV promoter (BLVp) or CMV promoter (CMVp) of 1 × 10^6 ^stably transduced cells were measured during a 10 s period. Bars represent the arithmetic mean and variance of 10 experiments. *P < 0.05; **P < 0.001 determined by t-test.

To assess directly the effect of constitutive Tax expression on the BLV promoter and CMV promoter, BLV Tax was provided as a transgene to cells. As expected, Tax significantly enhanced BLV promoter activity but had no effect on CMV promoter activity (Fig. 4) inducing BLVp activity about 48-fold in D17 cells and 4-fold in primary B cells. Interestingly, we found that when BLV infected cells were transduced with the Tax transgene, the resulting increase in BLV promoter activity was a greater-than-additive enhancement of BLV infection and Tax transgene (Table 1). This effect could likely be caused by Tax expressed from the trangene upregulating expression of the entire BLV provirus, including Tax. The effect on the CMV promoter was not significant. Further, BLVp activity was enhanced in cell lines FLK (2-fold) and BL3.1 (4-fold) actively producing high-levels of BLV (Fig. 5).

**Table 1:** Percent of Basal Luciferase Expression

Promoter	D17+Tax	D17+BLV	D17+Tax+BLV
BLVp	115 ± 7	1226 ± 15	2038 ± 202
CMVp	96 ± 5	130 ± 23	118 ± 14

### Trichostatin A non-specifically enhances BLV promoter and CMV promoter Activity

**Figure 6:**
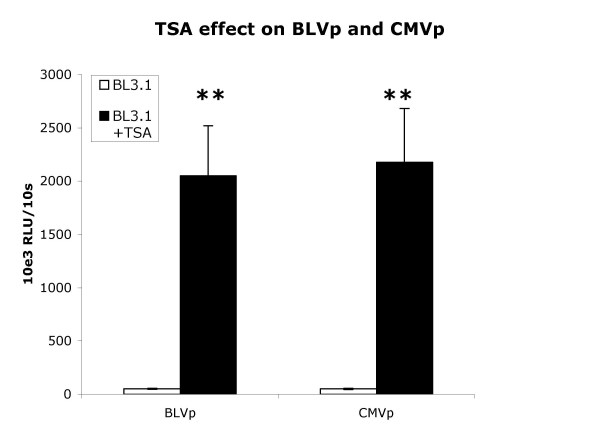
Trichostatin A (TSA) enhances BLVp and CMVp activity. Relative light units (RLU) of luciferase activity driven by either the BLV promoter (BLVp) or CMV promoter (CMVp) of 1 × 10^6 ^stably trasduced cells was measured during a 10 s period. BL3.1 cells were either non-treated or treated with 500 nM TSA for 48 h. Bars represent the arithmetic mean and variance of 10 experiments. **P < 0.001 determined by t-test.

The deacetylase inhibitor trichostatin A (TSA) has been shown to be the most efficient activator of BLV expression known to date [23]. To determine whether increased promoter activity due to TSA was a generalized attribute applicable to the CMVp as well, D17, FLK, and BL3.1 cells possessing BLVp or CMVp driven luciferase expression were treated with TSA. However, since CMVp activity was already 50-fold greater than BLVp activity in D17 cells, comparison of TSA induced promoter activities in a cell line where BLV and CMV promoter activities were similar would permit a more effective evaluation of TSA on the two promoters. Further, TSA induced much less death within the 48 h assay period in BL3.1 cells compared to other cell lines tested (< 10%). Using BL3.1 with counts adjusted for live cells, TSA treatment enhanced both BLVp and CMVp activity by about 40-fold (Fig. 6) indicating TSA was a non-specific promoter enhancer.

## Discussion

Viral promoters are used in mammalian expression vectors because they can have strong activity in a wide variety of cells of differing tissues and species. Probably the most employed is the CMV promoter because of its proven high-level constitutive expression in a variety of mammalian cell lines [24,25]. While constitutive transgene expression is suitable for certain research or gene therapy applications, a strong regulated transgene expression is preferable in many other applications [26]. The BLV promoter, consisting of the U3 region of the LTR, is highly dependant upon Tax for activation and transgene expression. In this study, we set out to determine the strength of BLV promoter activity compared to the strength of the CMV promoter to ascertain the utility of the BLV promoter for mammalian expression vectors. Information on the BLV promoter describing the *cis*-acting elements and the dependence upon Tax using reporter vectors in mammalian cell lines has been published [13,27,28]. However, a direct comparison of promoter strength of the BLV promoter and the standard of mammalian expression vectors, the CMV promoter, has not been performed.

Several attributes are important in developing a mammalian expression vector. Probably the most important attribute of a mammalian expression vector promoter is its ability to accomplish high-level transcriptional activity in a large variety of cell types of different tissues and species. Our studies showed that the BLV promoter could achieve similar high-level activity to the CMV promoter in cells expressing BLV Tax or infected with BLV. This comparatively high BLV promoter activity was demonstrated in D17 cells which we have found to be the highest expresser of CMV promoter driven transgenes of all cell lines tested in our laboratory. The CMV promoter activity was still about 5-fold greater than BLV promoter activity in the BLV infected D17 cells compared to the relatively equal activity of the CMV promoter versus BLV promoter in BLV infected FLK cells. However, FLK cells contain four copies of the BLV provirus [29] whereas BLV infected D17 cells contain a single copy of the provirus (data not shown). Thus there may be relatively greater expression of Tax in FLK cells effecting greater activity of the BLV promoter. Quantitative levels of Tax in BLV infected D17 or FLK cells were not measured. In this study, we showed relative to the CMV promoter high levels of induced BLV promoter activity in cell lines of canine osteosarcoma (D17), fetal lamb kidney (FLK), bovine B-lymphosarcoma (BL3.1), and bovine primary B cell origin. We also have data (not shown) demonstrating high BLV promoter driven transcriptional levels in cell lines derived from bat lung (TB1Lu), monkey kidney (Vero), and human kidney (HEK-293). Other researchers have also shown high BLV promoter activity using reporter gene assays in cell lines of various tissues derived from cow, dog, cat, mouse, human, monkey, sheep, and hamster [5,6,13,22,23,27,28]. Clearly the BLV promoter possesses the significant trait of high-induced expression in a wide variety of cell types.

A second important attribute of an inducible promoter apart from high-induced expression is low basal expression. Researchers have reported barely detectable BLV promoter Tax-independent activity through luciferase assays of COS-1, C8, and KU-1 cell transient transfections [28]. Our results using reporter vector stable D17 cell lines showed low but definite BLV promoter basal activity. Others measuring BLV promoter-driven luciferase activity in transiently transfected D17 cells reported an above background activity of the BLV promoter, but the basal activity seemed much closer to background than we report here [6,23]. The difference could be due to vectors employed (the commercial retrovector we used had weak promoter activity from the 5'LTR (data not shown) and our vectors contained the WPRE), or that we used stable cell lines versus transient transfections. Researchers using B cell lines (Raji, Daudi, DG75, A20) also showed low, but definite BLV promoter activity in transient and stable transfected cells similar to our results using primary B cells [6,13,27]. Nevertheless, in all of these studies Tax addition was able to induce expression ranging from 50 to 800-fold over basal expression. Our data showed Tax enhanced BLV promoter activity to levels comparable to the CMV promoter. A low but significant BLV promoter Tax-independent activity is not surprising considering the E boxes, CRE, GRE, NFkB and PU.1/Spi-B binding sites are available for cellular transactivating factors (Fig. 1). In fact, mutation studies of these *cis*-elements have demonstrated significant decreases in basal level activity, as with the mutation of the GRE site [30], significant increases in basal level activity, as with the mutation of the CRE sites [6], or either decrease or increase in basal activity, depending on the cell line assayed, as with mutations of the E box [30]. Still, compared to CMV promoter activity, or Tax-induced activity, BLV promoter basal activity is very low.

A third important attribute for an inducible promoter would be a sensitive modulated response to a specific inducer. Enhancement of the BLV promoter can occur independent of Tax by the addition of activating agents. Phorbol esters, phytohemaglutinin, and lipopolysaccharides have all been shown to enhance BLV promoter expression [31]. However, all of these agents are non-specific activators and upregulate many promoters within the cell [32]. The most efficient activator of BLV expression is the deacetylase inhibitor, trichostatin A (TSA). Addition of TSA to D17 cells enhanced luciferase expression driven by the BLV promoter 11-fold over basal expression [23]. In BL3.1 cells, less variability occurred from TSA induced cell death and basal BLVp and CMVp activity was relatively the same. TSA upregulated activity of both BLV and CMV promoters within BL3.1 by about 40-fold. In contrast, the BLV promoter was specific to Tax activation, while CMV promoter expression was not affected by Tax. For example in D17 cells, Tax specifically increased BLVp activity 48-fold.

Nevertheless, the transactivating properties of BLV Tax are not limited to activation of the BLV promoter. Tax has been shown to upregulate Bcl-2 and increase nuclear NFkB activity [17]. Tax expression induces immortalization of primary rat embryo fibroblasts and causes cytokine-independent B cell growth [17,33]. These "side effects" of Tax may deter the use of BLV promoter for mammalian expression vectors. However, studies have demonstrated that the BLV promoter transactivation and immortalization activities of wild-type Tax can be dissociated by mutations within specific regions of the protein [9]. In fact, phosphorylation of Tax serines 106 and 293 are required for in vitro cell transformation but not BLV LTR transactivation [34]. Tax transcriptional activity requires an amino-terminal zinc finger and an internal leucine-rich activation domain [9]. Phosphorylation-deficient Tax mutants have been developed [33] and could be used in place of wild-type Tax for BLV promoter transactivation. Other mutations of Tax were shown to enhance BLV promoter activity in 293T cells by 10-fold over wild-type Tax [22]. However this mutant also transactivated the cellular proto-oncogene c-*fos*. Clearly, there is great potential to magnify the desirable traits of the BLV promoter/Tax system for mammalian expression vectors and minimize undesirable traits.

## Conclusions

To determine whether the BLV promoter could be a useful mammalian expression vector element, we compared its activity with the CMV immediate early promoter in dog osteosarcoma (D17), BLV-infected fetal lamb kidney (FLK), BLV-infected bovine B-lymphosarcoma (BL3.1), and primary bovine B-cells. Without concomitant Tax expression from a transgene or BLV infection, the BLV promoter activity was low compared to CMV promoter activity. In the presence of Tax or BLV expression, the BLV promoter activity became equally as active as the CMV promoter. The CMV promoter was not influenced by Tax or BLV. Tax overexpressed as a transgene in BLV infected cells resulted in BLV promoter expression greater than CMV promoter expression. The deacetylase inhibitor, trichostatin A was a potent upregulator of both BLV and CMV promoters. Our results indicate the BLV promoter has great potential use as an inducible promoter for mammalian expression vectors.

## Competing interests

None declared.

## Authors' contributions

JSH carried out cell culture work including transfection/transduction and luciferase assays, data preparation and analyses, and drafted the manuscript. KAE performed genetic engineering of vectors. LSK did preliminary work to establish study concepts. RDB and GAS participated in the design and coordination of the study. All authors read and approved the final manuscript.
